# The Cerebrovascular Basement Membrane: Role in the Clearance of β-amyloid and Cerebral Amyloid Angiopathy

**DOI:** 10.3389/fnagi.2014.00251

**Published:** 2014-09-19

**Authors:** Alan W. J. Morris, Roxana O. Carare, Stefanie Schreiber, Cheryl A. Hawkes

**Affiliations:** ^1^Faculty of Medicine, Clinical and Experimental Sciences, University of Southampton, Southampton, UK; ^2^Department of Neurology, Otto-von-Guericke University, Magdeburg, Germany; ^3^German Center for Neurodegenerative Diseases (DZNE), Helmholtz Association, Magdeburg, Germany

**Keywords:** Alzheimer’s disease, cerebral amyloid angiopathy, perivascular drainage, basement membrane, β-amyloid

## Abstract

Cerebral amyloid angiopathy (CAA), the accumulation of β-amyloid (Aβ) peptides in the walls of cerebral blood vessels, is observed in the majority of Alzheimer’s disease (AD) brains and is thought to be due to a failure of the aging brain to clear Aβ. Perivascular drainage of Aβ along cerebrovascular basement membranes (CVBMs) is one of the mechanisms by which Aβ is removed from the brain. CVBMs are specialized sheets of extracellular matrix that provide structural and functional support for cerebral blood vessels. Changes in CVBM composition and structure are observed in the aged and AD brain and may contribute to the development and progression of CAA. This review summarizes the properties of the CVBM, its role in mediating clearance of interstitial fluids and solutes from the brain, and evidence supporting a role for CVBM in the etiology of CAA.

## Alzheimer’s Disease and Cerebral Amyloid Angiopathy

Alzheimer’s disease (AD) is a chronic progressive neurodegenerative disease, currently estimated to affect 35.2 million people worldwide, making it the commonest form of dementia (Alzheimer’s Disease International, 2014). Life expectancy after diagnosis ranges from 4 to 6 years (Larson et al., 2004). The main risk factor is age, with the chance of developing AD doubling every 5 years after the age of 65 years (Gao et al., 1998). Other non-modifiable risk factors include mutations in genes encoding the amyloid precursor protein (APP) and presenilin-1 and -2 in familial AD cases (Tanzi, 2012) and possession of the apolipoprotein E (ApoE) ε4 allele in sporadic AD (Tanzi, 2012). Midlife hypertension, diabetes, and hypercholesterolemia are modifiable risk factors that increase the risk of developing AD in late life (Launer et al., 2000; Shah et al., 2012).

Alzheimer’s disease is characterized clinically by a slow and gradual deterioration in cognitive function as well as psychiatric symptoms and behavioral disturbances such as depression, delusions, and agitation (Burns, 1992; Bird and Miller, 2007). Neuropathologically, AD is associated with brain region-specific neuronal loss and synaptic degeneration (Bondareff et al., 1982; Whitehouse et al., 1982; Vogels et al., 1990; Aletrino et al., 1992), which begin in the entorhinal cortex and advance to the hippocampus and posterior temporal and parietal cortices (Begley et al., 1990; Braak and Braak, 1991). The end result of this degenerative neuronal loss is brain shrinkage and ventricular enlargement.

Two classical pathological features of AD are the accumulation of neurofibrillary tangles (NFTs) and senile plaques (Braak and Braak, 1991; Alafuzoff et al., 2008). NFTs are formed predominantly of ubiquitinated hyperphosphorylated tau (Esiri et al., 2004). Tau is a microtubule-associated protein that is present in mature neurons. In AD, tau becomes hyperphosphorylated and aggregates into NFTs of paired helical filaments (Iqbal et al., 2010). These aggregated bundles are seen pathologically as intraneuronal tangles in dystrophic neurites surrounding senile plaques and in the neuropil as neuropil threads (Braak et al., 1986; Grundke-Iqbal et al., 1986). Senile plaques are formed predominantly of amyloid β (Aβ). Amyloidogenic Aβ is produced from the sequential cleavage of APP by two proteases, β- and γ-secretase.

In addition to its deposition in the parenchyma, Aβ accumulates in the walls of cerebral blood vessels as cerebral amyloid angiopathy (CAA) (Miyakawa et al., 1982; Vinters, 1987; Weller et al., 2008). CAA is observed in 30–40% of non-demented elderly individuals and 60–95% of AD brains studied at autopsy (Vinters, 1987; Castano and Frangione, 1988; Haan et al., 1991; Jellinger, 2002). It affects predominantly leptomeningeal and cortical arteries but also occurs in cerebral capillaries (Vinters, 1987; Giannakopoulos et al., 1997; Roher et al., 2003). Unlike the parenchymal senile plaques that are composed principally of Aβ42 (Dickson et al., 1988), vascular Aβ deposits comprise predominantly Aβ40 (Suzuki et al., 1994). CAA is associated with capillary thinning and vessel tortuosity, inhibition of angiogenesis, and the death of pericytes, endothelial, and smooth muscle cells (Perlmutter et al., 1994; Miao et al., 2005; Haglund et al., 2006; Tian et al., 2006; Burger et al., 2009). Cerebral hypoperfusion, microhemorrhages, and cognitive impairment have also been shown to be associated with CAA severity (Natte et al., 2001; Pfeifer et al., 2002a,b; Shin et al., 2007; Chung et al., 2009).

The origin of the vascular Aβ deposits has been contested since the early twentieth century when it was first proposed that blood-borne Aβ was causing the phenomenon (Scholz, 1938). In the presiding half-century, other mechanisms were suggested, including the production of Aβ by vascular smooth muscle cells (VSMCs) (Wisniewski and Wegiel, 1994; Vinters et al., 1996; Wisniewski et al., 1996). However, the dichotomy between the specific localization of CAA to cerebral blood vessels and the ubiquitous expression of VSMCs throughout the body, suggested a brain-based origin of vascular Aβ. This hypothesis was supported by findings that transgenic mice producing mutant human Aβ only in the brain develop CAA (Herzig et al., 2004, 2006).

Based in part on electron microscopy studies showing the deposition of Aβ within the cerebrovascular basement membrane (CVBM) of the arterial tunica media (Wisniewski and Wegiel, 1994), Weller et al. (1998) hypothesized that parenchymal Aβ is normally cleared from the brain along CVBM and that CAA results from the failure of this system in the aged brain.

This review will summarize the properties of the CVBM, its role in mediating clearance of interstitial fluid (ISF) and solutes from the brain, and evidence supporting a role for CVBM in the etiology of CAA.

## The Basement Membrane of the Brain

**Figure 1:**
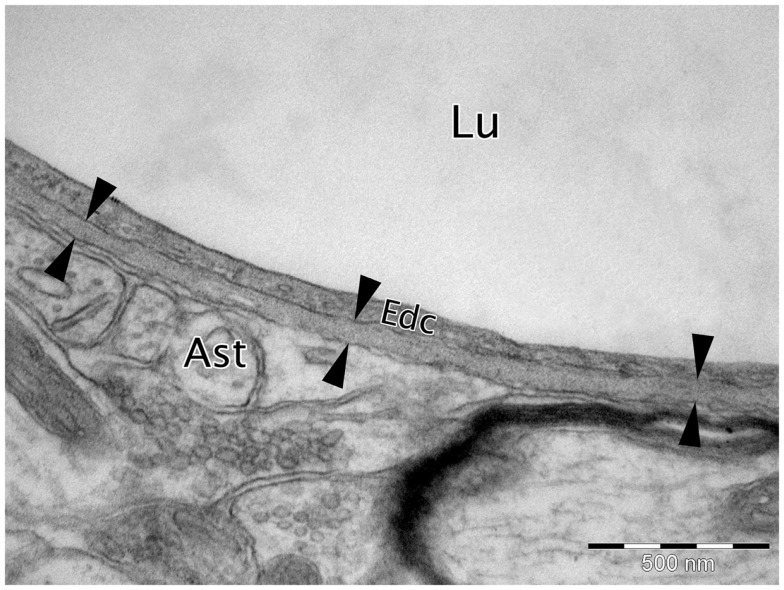
**Micrograph of cortical Wistar Kyoto rat capillary**. The cerebrovascular basement membrane is the electron dense area between the six arrow heads. Lu, capillary lumen; Edc, endothelial cell; Ast, astrocyte end foot. TEM, 50,000×, scale bar, 500 nm.

Basement membranes (BMs) are specialized extracellular matrices 50–100 nm in size when visualized by transmission electron microscopy (TEM) that cover the basal aspect of all endothelial and epithelial cells and surround fat, muscle, and Schwann cells (Carlson et al., 1978; Yurchenco and Schittny, 1990; Weber, 1992; Timpl, 1996; Miner, 1999; Candiello et al., 2007; McKee et al., 2009; Rasi et al., 2010) (Figure 1). They are formed from a variety of intracellularly produced proteins that are secreted into the surrounding extracellular matrix (ECM). BMs provide structural support to tissues, separate cells from connective tissue, and are important in modulating cellular signaling pathways (Yurchenco and Schittny, 1990; Timpl and Brown, 1996; Kalluri, 2003). The majority of BMs contain the following proteins; type IV collagen, laminins, nidogen, and the major heparan sulfate proteoglycan (HSPG) perlecan (Timpl and Brown, 1996; Halfter et al., 2013). Each of these core BM proteins has a subset of isoforms, there are 6 isoforms of type IV collagen, 2 nidogen isoforms, 2 major HSPGs (perlecan and agrin), and 16 different isoforms of laminin (Sorokin, 2010). Variation in the structural composition of the core BM proteins results in protein heterogeneity. For example, collagen IV comprises six distinct polypeptide α-chains [α1(IV)–α6(IV)], which assemble with high specificity to form three distinct isoforms, α1α1α2, α3α4α5, and α5α5α6 (Khoshnoodi et al., 2008). Laminins are composed of α, β, and γ polypeptide chain (Miner and Yurchenco, 2004). Currently 5 distinct α chains, 3 distinct β chains, and 3 distinct γ chains have been characterized and they assemble in various combinations to form 16 different laminin isoforms, which are classified as α1β1γ1, which is abbreviated to 111, depending on which chains make up the final molecule (Aumailley et al., 2005). Auxiliary BM proteins including, type XV collagen, type XVIII collagen, agrin, osteonectin [also known as secreted protein acidic and rich in cysteine (SPARC) or BM protein 40 (BM40)], BM90, and fibulin contribute to the structural and functional diversity of the BM across different tissues (Timpl, 1996).

In the brain, BM proteins are expressed in the ECM of the parenchyma and within multiple layers of the cerebral blood vessels. Collagen IV (Khoshnoodi et al., 2008), laminin (Hagg et al., 1989; Hunter et al., 1992; Jucker et al., 1996a,b; Hallmann et al., 2005), nidogen -1 and -2 (Bader et al., 2005), HSPGs perlecan (Noonan and Hassell, 1993), fibronectin (Caffo et al., 2008), and agrin (Barber and Lieth, 1997), are the predominant BM constituents of the brain. However, there are variations in the composition of individual cerebral BMs, especially with regards to laminin. For example, the BM of the glia limitans contains both laminin α1 and α2 (Hallmann et al., 2005), whereas only laminin α1 is expressed in the meningeal epithelium and the astrocytic endothelium contains only laminin α2 (Hallmann et al., 2005). The different protein composition within each cerebral BM influences numerous cellular functions such as neural stem cell differentiation and migration, axon formation, apoptosis, myelination, and glial scar formation (Perris, 1997; Goldbrunner et al., 1999; Thyboll et al., 2002).

**Figure 2:**
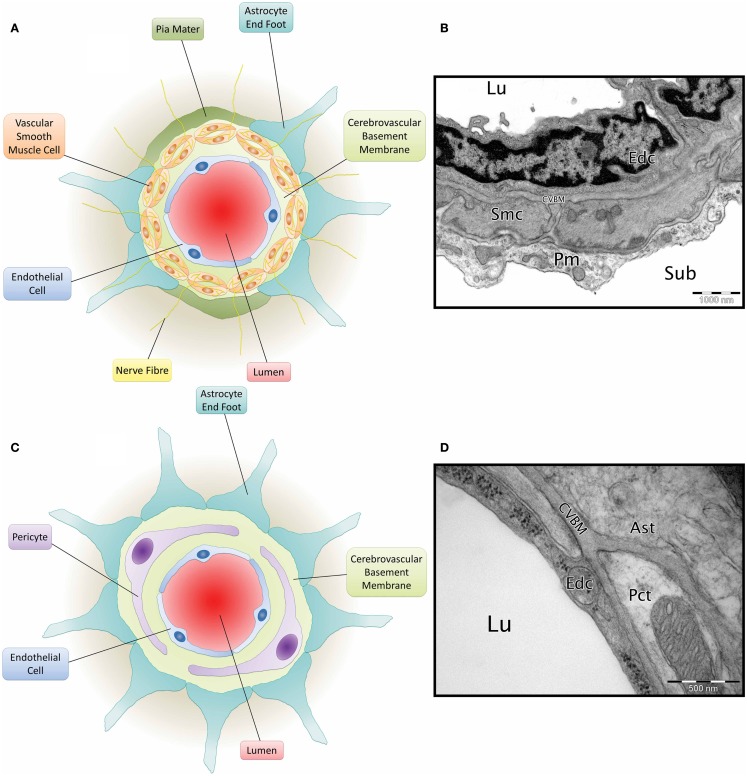
**Composition of the neurovascular unit**. **(A)** Diagram of leptomeningeal artery. Viewed from the center: arterial lumen (red), endothelial cell (blue), cerebrovascular basement membrane (light green), vascular smooth muscle cells (orange) have a cerebrovascular basement membrane running between them and are penetrated by nerve fibres (yellow), pia mater (dark green), and astrocyte end foot (teal). **(B)** Micrograph of C57BL/6 mouse cortical artery. Lu, lumen; Edc, endothelial cell; Smc, vascular smooth muscle cell; CVBM, cerebrovascular basement membrane; Pm, pia mater; sub, subarachnoid space. TEM, 25,000×, scale bar, 1000 nm, *image credit*: *Matthew M. Sharp*. **(C)** Diagram of a cerebral capillary. Viewed from the center; capillary lumen (red), endothelial cell (blue), cerebrovascular basement membrane (light green), pericyte (purple), and astrocyte end foot (teal). **(D)** Micrograph of Wistar Kyoto rat cerebral capillary. Lu, lumen; Edc, endothelial cell; Pct, pericyte; CVBM, cerebrovascular basement membrane; Ast, astrocyte end foot. TEM, 50,000×; scale bar, 500 nm.

Cerebrovascular basement membranes play an important role in blood vessel development and health, formation and maintenance of the blood–brain barrier (BBB) and migration of peripheral cells including leukocytes into the brain (Sixt et al., 2001; Zlokovic, 2008; Wu et al., 2009). Endothelial cells, astrocytes and smooth muscle cells (Zlokovic, 2008) all contribute to the composition of the CVBM (Yousif et al., 2013) and this also receives contributions from pericytes (Dohgu et al., 2005; Bell et al., 2010) (Figure 2). The CVBM comprises collagen IV, laminin, perlecan, and nidogen as well as several minor glycoproteins (Hallmann et al., 2005). There is, however, variation in the expression of laminin isoforms within the CVBM of different vessel types (Sorokin, 2010). At the capillary level, the BM is fused between the endothelial cells and astrocyte end feet and contains laminin α2, α4, and α5 (Hallmann et al., 2005). Cerebral arterioles and arteries contain an endothelial BM (containing laminin α4 and/or α5), a BM that surrounds the VSMCs within tunica media (containing laminin α1 and α2) and an astrocytic BM (containing laminin α1 and α2) (Sixt et al., 2001). Veins comprise an endothelial BM (containing laminin α4 and a patchy distribution of laminin α5), a smooth muscle BM (containing laminin α1 and α2), and an astrocytic BM (containing laminin α2, α4, and α5) (Sixt et al., 2001; Yousif et al., 2013).

## Perivascular Elimination Pathway of ISF

The brain only accounts for around 2% of total body mass, but it is responsible for approximately a quarter of the body’s total oxygen and glucose consumption (Zlokovic, 2008). This metabolic output produces large quantities of waste neurotoxins that must be transported, along with other solutes, out of the brain for eventual excretion from the body. Unlike peripheral organs that communicate with the lymphatic system to remove ISF, the brain does not have conventional lymphatics. Solutes that cannot be eliminated across the BBB or into the CSF must therefore be removed via alternative routes.

**Figure 3:**
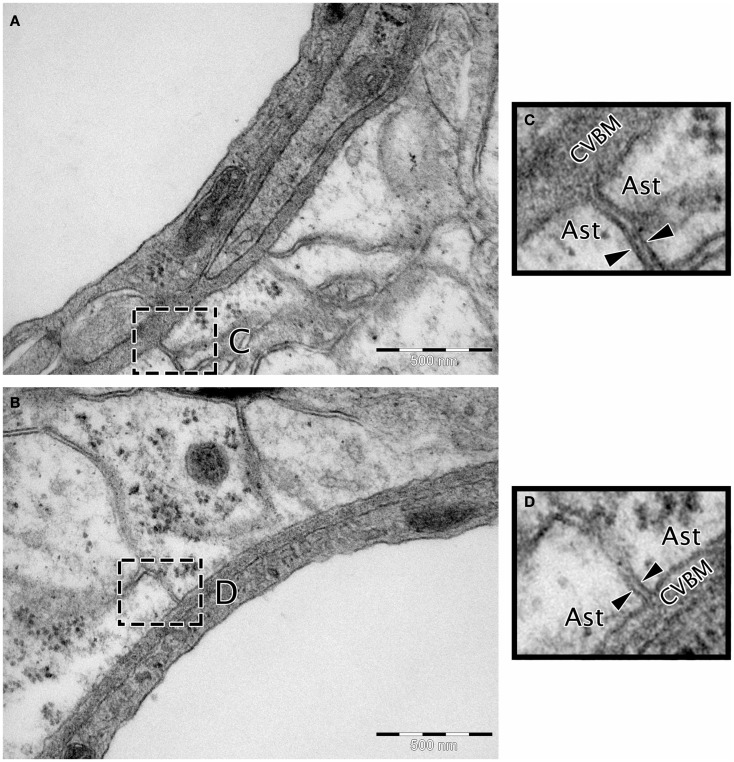
**Micrographs showing the continuity between the extracellular matrix and the cerebrovascular basement membrane**. **(A,B)** Wistar Kyoto rat cerebral capillary, TEM, 50,000× scale bar 500 nm. **(C,D)** Magnified insert highlighting continuity between the BM and extracellular matrix. The extracellular matrix is the electron dense area between the two arrow heads in **(C,D)**. Ast, astrocyte end foot; CVBM, cerebrovascular basement membrane.

Transmission electron microscopy analyses indicate continuity between the extracellular spaces of the brain and the endothelial capillary BM (Figure 3), suggesting a putative pathway by which parenchymal ISF may be cleared. Results from early drainage studies found that large molecular weight compounds injected into the brain parenchyma spread along perivascular spaces and could be observed in the walls of leptomeningeal arteries and in cervical lymph nodes (Szentistvanyi et al., 1984). Further, the clearance of small and large molecules occurred at similar rates (Cserr et al., 1981), suggesting that movement of ISF was dependent on bulk flow rather than diffusion, with an estimated clearance rate of 0.15–0.29 μL/min/g in the rat brain and 0.10–0.15 μL/min/g in the rabbit (Szentistvanyi et al., 1984; Abbott, 2004; Carare et al., 2008).

The role of the CVBM in the perivascular clearance of parenchymal solutes was shown experimentally by Carare et al., who found that fluorescent tracers were localized to the BM of both capillaries and arteries within 5 min of injection into the deep gray matter (Carare et al., 2008). In arterial walls, the tracers were specifically located in the BM of the tunica media, but not in the endothelial BM or the outer most BM between the arterial wall and brain parenchyma (Carare et al., 2008). At 3 h after injection, the tracer had cleared the CVBMs and tracers were only visible within perivascular macrophages (Carare et al., 2008). More recent studies using multiphoton imaging have confirmed the drainage of intracerebrally injected tracers along perivascular BMs (Arbel-Ornath et al., 2013). This pathway appears specific to small solutes, as large molecules, including immune complexes, Indian ink, and 20 nm fluorospheres are unable to enter the CVBM (Zhang et al., 1992; Barua et al., 2012; Teeling et al., 2012).

The motive force for the perivascular elimination pathway is still unclear. It has been hypothesized that cerebral blood flow plays a major role because clearance ceases immediately after cardiac arrest (Carare et al., 2008). Mathematical models have also provided insight into potential mechanisms by highlighting the potential for reflection wave driven flow (Schley et al., 2006; Wang and Olbricht, 2011). Each arterial pulsation is followed by a contrary (reflection) wave that passes along the vessel wall in the opposite direction to blood flow, which matches the observed pattern of distribution of solutes injected into the gray matter (Schley et al., 2006; Wang and Olbricht, 2011). This reflection wave could be aided by conformational changes in the CVBMs occurring during constriction and relaxation of the vessel wall in arteries (Carare et al., 2013) and of pericytes in capillaries. BM proteins may also adopt a valve-like configuration to prevent the ISF back flow (Schley et al., 2006).

## Role of CVBM and Perivascular Drainage in CAA

**Figure 4:**
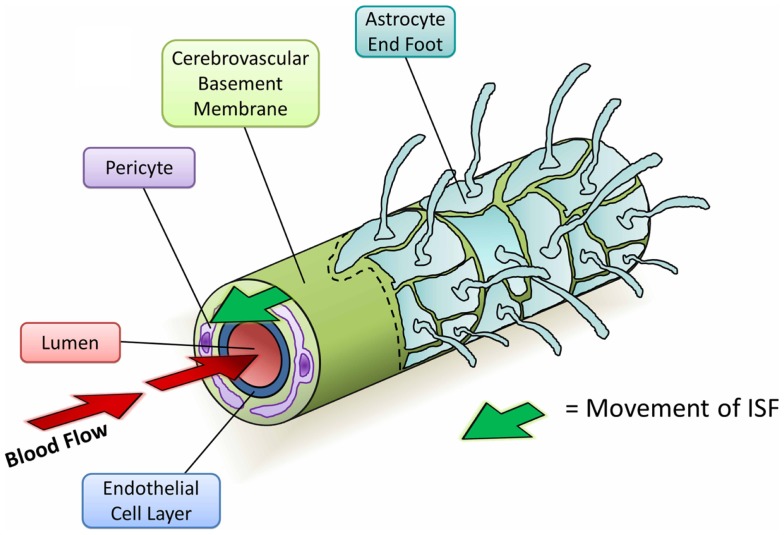
**Diagram depicting the perivascular elimination of solutes along the cerebrovascular basement membrane of a cerebral capillary**. The arterial pulsatile wave driving blood flow into the brain (red arrows) is followed by a refractory wave, which may drive the movement of interstitial fluid (ISF) out of the brain along the cerebrovascular basement membrane (green arrow). Conformational changes in cerebrovascular basement membranes during the refractory wave may provide a valve-like mechanism that promotes unidirectional flow of ISF. Viewed from the center: capillary lumen (red), endothelial cell layer (blue), cerebrovascular basement membrane (light green), pericyte (purple), and astrocyte end foot (teal) with a section removed beyond dotted line.

Since the initial studies into perivascular elimination of solutes, it has been shown that soluble Aβ follows the same route after injection (Figure 4) (Hawkes et al., 2011, 2012, 2013). The pattern of distribution closely matches with that of human CAA, supporting the hypothesis that CAA outlines the natural route of elimination of cerebral Aβ. Multiple additional lines of evidence suggest an important role for CVBMs in the clearance of Aβ and its failure in CAA:

### Morphologic changes in the cerebrovasculature and CVBM

Thickening, splitting, duplication, and the presence of abnormal inclusions in the CVBM have been reported in the brains of aged animals and humans and to a greater degree in the AD brain (Perlmutter, 1994; Kalaria, 1996; Farkas and Luiten, 2001; Shimizu et al., 2009). Thickening of the CVBM in both rodents and humans appears to be most predominant in brain areas that are susceptible to AD and CAA pathology (Zarow et al., 1997; Hawkes et al., 2013) and precedes CAA onset in TGF-β transgenic mice (Wyss-Coray et al., 2000). Further, increasing age is associated with arterial rigidity, elongation, and tortuosity, as well as a reduction in the vascular content of smooth muscle (Dahl, 1976). Such changes may diminish the force of arterial contraction and thereby diminish the driving force for perivascular clearance of Aβ.

### Changes in the biochemical composition of the CVBM

Laminin, nidogens, and collagen IV inhibit the aggregation of Aβ and destabilize pre-formed fibrils of Aβ *in vitro*, while HSPG agrin and perlecan accelerate its aggregation (Aumailley and Krieg, 1996; Bronfman et al., 1996, 1998; Castillo et al., 1997; Cotman et al., 2000; Kiuchi et al., 2002a,b). Decreased amounts of collagen IV have been reported in small diameter vessels (<50 μm) from AD patients compared to aged-matched controls (Christov et al., 2008). Conversely, the levels of HSPGs were increased in AD brains (Berzin et al., 2000; Shimizu et al., 2009). Similar alterations have also been observed in the levels of collagen IV, laminin, nidogen 2, fibronectin, and perlecan in CAA-vulnerable brain regions of aged mice (Hawkes et al., 2013). These data suggest a change in the BM composition of the aged brain toward a pro-amyloidogenic environment. In addition, mice expressing the human ApoE ε4 allele show greater changes in CVBM expression over the lifecourse than mice expressing human Apo ε3 allele, in association with disrupted perivascular drainage of Aβ40 (Hawkes et al., 2012).

### Relationship between parenchymal and vascular Aβ

Increased CAA severity has been noted in the brains of both mice and humans following anti-Aβ immunotherapy in association with decreased cerebral plaque load (Pfeifer et al., 2002b; Wilcock et al., 2004; Patton et al., 2006; Boche et al., 2008). These vascular Aβ deposits contained high levels of Aβ42, suggesting that following immunization, parenchymal Aβ became solubilized, drained along CVBMs, and became entrapped in the perivascular drainage pathways.

Collectively, these findings indicate that alterations in the composition, level of expression, or morphology of CVBMs may disturb perivascular drainage of Aβ from the parenchyma and initiate the deposition of Aβ within the walls of the cerebral vasculature, leading to a feed-forward mechanism of Aβ accumulation as CAA.

## Conclusion

Increasing evidence supports a role for the cerebrovasculature in the development of AD. Preventative strategies targeting cardiovascular risk factors have been shown to be effective in reducing the incidence of AD (Guo et al., 1999; in’t Veld et al., 2001; Ohrui et al., 2004; Khachaturian et al., 2006; Haag et al., 2009a,b; Sink et al., 2009; Chuang et al., 2014), supporting the importance of maintaining cerebral vascular health across the lifespan in the prevention of disease. The CVBM plays an important role in cerebrovascular health, including mediating the clearance of Aβ. Therapeutic strategies targeted at improving and/or facilitating perivascular drainage of Aβ along the CVBM of the elderly brain may therefore represent a novel approach to the treatment of AD and CAA.

## Conflict of Interest Statement

The authors declare that the research was conducted in the absence of any commercial or financial relationships that could be construed as a potential conflict of interest.
